# Unilateral Vocal Cord Paralysis following Insertion of a Supreme Laryngeal Mask in a Patient with* Sjögren's* Syndrome

**DOI:** 10.1155/2016/8185628

**Published:** 2016-11-27

**Authors:** T. O. J. Masarwa, I. H. F. Herold, M. Tabor, R. A. Bouwman

**Affiliations:** Department of Anesthesiology, Intensive Care and Pain Medicine, Catharina Ziekenhuis, Eindhoven, Netherlands

## Abstract

Since its introduction in 1988 by Dr. Archie Brain, the laryngeal mask airway (LMA) is being used with increasing frequency. Its ease of use has made it a very popular device in airway management and compared to endotracheal intubation it is less invasive. The use of LMA was on the rise, so has been the incidence of its related complications. We report severe unilateral vocal cord paralysis following the use of the supreme laryngeal mask (sLMA) in a patient with* Sjögren's* syndrome. In addition, we propose possible mechanisms of injury, review the existing case reports, and discuss our findings.

## 1. Introduction

The laryngeal mask airway (LMA) was first introduced into practice by Dr. Archie Brain in 1988 who used plaster casts made from cadaveric pharynges. The LMA became quickly a very popular device due to its ease of placement and being less invasive compared to endotracheal intubation [1]. Over the years several modifications have been brought into the LMA to make it easier to be inserted and to minimize pharyngolaryngeal complications such as sore throat, vocal cord palsy, arytenoid dislocation, and tongue cyanosis. The supreme LMA (sLMA) has proven to be a safe and efficacious supraglottic airway device compared to the ProSeal (pLMA) and classical LMA (cLMA) [2–4]. However, we encountered a case of hoarseness after insertion of a sLMA in a patient with* Sjögren's* syndrome. Although there have been several case reports of vocal cord paralysis following the use of the cLMA and pLMA [5–18], there have been no clinical reports following insertion of a sLMA. Increased cuff pressure and distention of the hypopharynx with subsequent direct pressure on the recurrent laryngeal nerve are cited as a possible mechanism of injury. In this report we describe a case in which a patient with* Sjögren's* syndrome developed a unilateral recurrent laryngeal neuropraxia after sLMA insertion. The possible pathogenetic mechanisms are also discussed.

## 2. Case Description

A 69-year-old female (160 cm, 50 kg, ASA I) was scheduled to undergo a vaginal prolapse operation. Her medical history included* Sjögren's* syndrome and glaucoma. In addition to treatment with timoptol, she occasionally used omeprazol due to gastric complaints. Preoperative physical evaluation showed a normal mouth opening and Mallampati score III with limited neck mobility; a sLMA was chosen to maintain the airway preoperatively. Anesthesia was induced with 15 mcg sufentanil and 300 mg propofol intravenously. A size 4 sLMA was inserted using the standard technique. Insertion of the sLMA was performed by a second-year resident in one attempt. Insertion was without any difficulty and was done under supervision of a Staff Anesthesiologist. After insufflation with about 20 mL air the seal allowed ventilation without leakage. Although cuff pressure is routinely measured in our hospital, in this case it was accidently omitted and not recorded. An inspiratory airway pressure of 18 cm H_2_O was obtained and maintained throughout the whole operation. The cuff volume was not noted at that time, and the intracuff pressure was not measured on sLMA insertion. Ventilation was set to a 50/50 oxygen/air mixture, ventilatory frequency of 12 bpm, and inspiratory : expiratory ratio of 1 : 2. General anesthesia was maintained with sevoflurane in 50% oxygen. The surgery was completed uneventfully as ventilation and blood pressure were adequate throughout the whole operation. During emergence no complications occurred and total anesthesia time was 1 h and 5 minutes.

**Figure 1:**
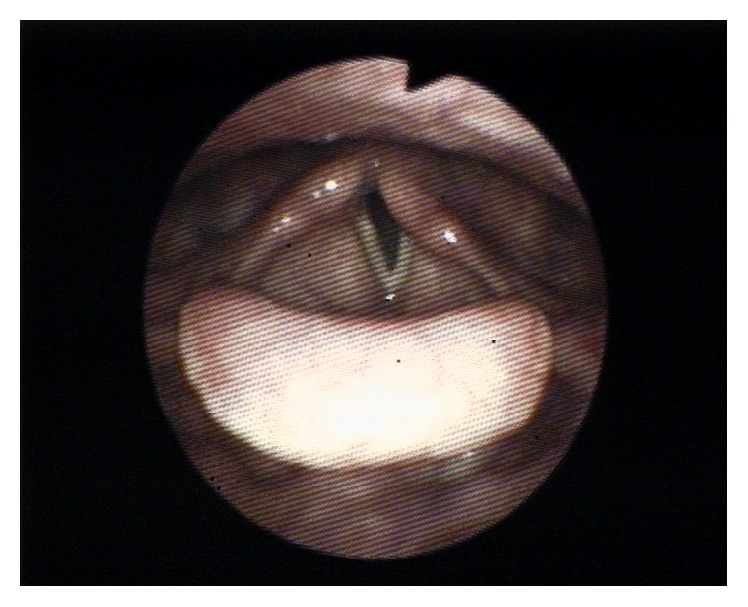
Left vocal cord paralysis during phonation as seen during indirect laryngoscopy, with permission from patient and Dr. Tabor.

Two days postoperatively the patient developed hoarseness and two weeks later she consulted an ENT specialist at our hospital as the complaint got worse. Fiberoptic laryngoscopy showed left vocal cord paralysis and left recurrent laryngeal nerve neuropraxia was diagnosed (Figure 1). A computed tomography (CT) of the neck and thorax showed no pathological lymphadenopathy or any tumors in the neck or the lungs. Blood analysis showed normal values. The findings were discussed with the patient and she was directed to a logopedist for voice therapy and an appointment was made over 6 weeks.

## 3. Discussion

As far as we know, this is the first case report showing the occurrence of vocal cord paralysis after the use of a sLMA. To date, there have been 14 case reports regarding recurrent laryngeal nerve injury after the use of a LMA. In all of these case reports a cLMA or pLMA were used [5–18]. The most common causes for vocal cord paralysis are (1) viral infection, (2) tumor, and (3) injury during surgery. If injury to the recurrent laryngeal nerve occurs after insertion of a LMA, then the most probable mechanism of injury is arytenoid dislocation or injury to the nerve due to direct compression. Other rare pathogenic mechanisms are local inflammation or hyperextension of the neck.

The recurrent laryngeal nerve is a branch of the vagus nerve and supplies all intrinsic muscles of the larynx, with the exception of the cricothyroid muscles. Both recurrent laryngeal nerves, right and left, emerge from the vagus nerve at the level of the arch of the aorta. On the right, it turns upward around the subclavian artery, whereas on the left it loops under the arch. Both nerves turn upward and enter the larynx near the apex of the pyriform sinus and behind the articulation of the thyroid and cricoid cartilages [19].

When correctly positioned, the tip of the LMA lies at the inferior border of the hypopharynx. The lateral edges of the cuff lie in the pyriform sinuses. When the cuff is inflated it tends to move 1,5 cm rostrally.

The insertion technique described by Dr. Brain is based on the physiology of swallowing and was intended to be easily reproduced. The head of the patient is placed in sniffing position while the jaw is held open gently. A deflated LMA of a proper size is advanced along the hard palate using the index finger over to guide it over the back of the tongue. The LMA is advanced until resistance is felt and it should not be held down when the cuff is being inflated. Once the cuff is inflated the LMA occupies its natural position [20].

The point at which the recurrent laryngeal nerve would be injured is along the pyriform sinuses where it enters the larynx. At this point the LMA is in close contact with the mucosa. Excessive cuff pressure can cause injury to the mucosa [21]; therefore the volume values suggested by the manufacturer should be applied. A previous study showed that even “normal” values can result in cuff pressure which may exceed the maximum suggested pressure (20–30 cm H_2_O), and maximum pressure should not exceed 44 cm H_2_O [2]. This may be due to gas expansion according to the law of Charles in which body temperature (37° Celsius) can warm up the air within the cuff from room temperature (20° Celsius) to body temperature. Measurement of intracuff pressure in any LMA should be done with a standard of care as it reduces postoperative laryngopharyngeal adverse events [2]. Furthermore it is known that nitrous oxide can increase intracuff volume up to 50% by way of diffusion [22].

Taking all of the above-mentioned factors into consideration, it is still difficult to point out the exact cause of nerve injury in our patient. She developed hoarseness only two days after surgery and not immediately; usually symptoms of neuropraxia are seen immediately after the injury took place. We used viscous lidocaine jelly to facilitate the insertion of the LMA. This may cause temporary vocal cord paralysis; this transient paralysis might be prolonged as lidocaine jelly dissolves poorly. We know that the use of lubricants containing lidocaine jelly is not recommended for the use of the LMA as stated clearly in the manual of instruction (Teleflex®). Unfortunately this was the only lubricant available at the time on the OR. Considering the fact that lidocaine has a very short half-life and the patient had no allergies to lidocaine we thought that the use of it would be without any serious adverse events. The vocal cord paralysis is unlikely in this case to be caused by the lidocaine as our patient developed hoarseness two days postoperatively and not immediately after emergence. Shortly after this incident we adjusted our policy in the OR, as we started using only lubricant for insertion of the LMA which does not contain any lidocaine. Unfortunately we cannot say whether we used excessive intracuff pressure. Although cuff pressure is routinely measured in our hospital, in this case it was accidently omitted and not recorded. Furthermore we cannot exclude a viral infection which is still the most common cause for recurrent laryngeal nerve neuropraxia. The prognosis in case of a viral infection is difficult to predict. Our patient had* Sjögren's* syndrome in which patients can experience severe dry oral mucosas causing hoarseness. However, the patient never complained of hoarseness in the past. We can also exclude hyperextension of the neck as a cause of the injury, because she had limited neck mobility. The real cause of her neuropraxia will still remain a matter of speculation.

In conclusion, recurrent laryngeal nerve injury after insertion of a LMA is a very rare complication. However it is advisable to know the standards for LMA size selection, not to overinflate the cuff to achieve a good seal, to monitor cuff pressure preoperatively, and to exclude first any other causes of vocal cord paralysis.
